# The Use of Social Media in Orthopedic and Trauma Surgery Education: A Cross-Sectional Survey of German-Speaking Residents and Medical Students

**DOI:** 10.3390/healthcare12202016

**Published:** 2024-10-10

**Authors:** Sebastian Schmidt, Ali Darwich, Sebastian Leutheuser, Daniel Krahl, Luis Navas

**Affiliations:** 1 Department of Orthopaedic and Trauma Surgery, University Medical Centre Mannheim, Medical Faculty Mannheim, University of Heidelberg, Theodor-Kutzer-Ufer 1-3, 68167 Mannheim, Germany; 2 Department of Orthopedic Surgery and Sports Traumatology, Witten/Herdecke University, Sana Medical Center, Aachener Str. 445-449, 51109 Cologne, Germany; 3 Department of Orthopedic Surgery, Vincentius-Diakonissen-Kliniken gAG, Steinhauserstraße 18, 76135 Karlsruhe, Germany; 4 Department of Orthopedic and Trauma Surgery, Orthopädische Klinik Paulinenhilfe, Diakonieklinikum, Rosenbergstrasse 38, 70192 Stuttgart, Germany

**Keywords:** social media, healthcare, residents, education, content

## Abstract

Background/Objectives: Social media has become a significant part of daily life, with platforms like Facebook and WhatsApp dominating usage. The COVID-19 pandemic further increased social media activity, including within the orthopedic community due to restrictions on physical gatherings. Despite the benefits of instant access to educational resources and interaction with experts, the lack of regulated editorial oversight on social media raises concerns about misinformation and privacy. This study aimed to evaluate the role of social media in orthopedic and trauma surgery education, focusing on platform use, user behavior, and engagement with educational content. Methods: A web-based survey was distributed to 912 residents and 728 medical students from the German-speaking Association for Arthroscopy and Joint Surgery (AGA) between June and July 2022. The questionnaire included 21 items covering demographics, platform use, activity patterns, engagement with educational content, and concerns about privacy. Results: Of the 339 respondents (129 medical students), 87% reported daily social media use, primarily via smartphones (93%). The most commonly used platforms were WhatsApp (84%), Instagram (68%), and YouTube (54%). About 26% of the content consumed was related to orthopedics or trauma surgery. While 70% engaged with specialist content by liking, commenting, or sharing, only 32% posted their own content. Additionally, 77% followed healthcare professionals or institutions, and 65% benefited from case presentations with images. Notably, 15% observed content that could violate patient privacy. Conclusions: Orthopedic residents and students are high-volume social media users but engage more passively with professional content. While most value educational material, concerns about privacy violations and inappropriate posts remain prevalent.

## 1. Introduction

A revolution in communication and information dissemination has taken place globally over the last two decades. In 2020 and 2021, a total of 3.9 and 4.26 billion people, respectively, used social media worldwide, showing an increase of 9% in users annually, a number projected to increase to almost six billion in 2027 [1]. Social networking is one of the most popular digital activities worldwide, and it is no surprise that social networking penetration across all regions is constantly increasing.

On average, internet users spend 144 min per day on social media and messaging apps, an increase of more than half an hour since 2015. Market leader Facebook was the first social network to surpass one billion registered accounts and currently boasts approximately 2.7 billion monthly active users, making it the most popular social network worldwide. In June 2020, the top social media apps in the Apple App Store included mobile messaging apps WhatsApp and Facebook Messenger, as well as the ever-popular app version of Facebook [2,3].

This popularity of social platforms has boosted access to journals and other educational material. This impact transcends the simple sharing of academic research. Today, it is feasible to share videos and technical content while simultaneously interacting and discussing it with subject matter experts. This real-time pooling of ideas and expertise has never been practicable before. Conversely, social media feeds are not controlled by the traditional strict editorial and peer-review processes and are vulnerable to the spread of false information or misinformation and violation of data privacy [4,5,6,7].

The popularity of social media in the orthopedic surgery community kicked up abruptly with the COVID-19 pandemic and the related social distancing policies that limited in-person meetings [8].

While the number of orthopedic residency programs actively using social media has increased year over year, social media is also increasingly being used for marketing and promotional purposes by both individual practitioners and institutions [9,10]. Residents indicate that social media presence has a positive impact on them and their choice of programs [11,12]. Additionally, studies in the US show that orthopedic residents use social media multiple times a day; however, these results are not generalizable due to local differences [9,11,13].

Therefore, this study aimed to create a survey to (1) assess social media platform use, (2) usage behavior and activity, as well as (3) educational content consumption.

## 2. Methods

A prospective online survey was conducted among orthopedic and trauma surgery residents and medical students interested in this field. The questionnaire was developed by the research team after reviewing the relevant literature and incorporating additional points of interest [11,14,15,16,17,18]. It was initially tested by five independent orthopedic and trauma surgeons, and their feedback was used to refine and finalize the questionnaire in order to ensure clarity and reliability. In accordance with current literature, which supports the use of nonvalidated and novel assessment tools for evaluating social media habits, this study applied such methods to assess the material presented on these platforms. The final questionnaire can be found in Supplementary Materials.

The survey included questions on demographics, social media platform use, usage behavior and activity, and consumption of educational content. Additional questions focused on the influence of social media in the selection of an employer and on privacy violations. All questions were closed questions and in 3 cases multiple answers were possible. The survey was created using SurveyMonkey software (http://www.surveymonkey.com; accessed 10 June 2020). The online questionnaire was sent together with a detailed purpose of the investigation and background information to all residents and medical students with membership in the German-speaking Society for Arthroscopy and Joint Surgery Residents Forum (AGA; Gesellschaft für Arthroskopie und Gelenkchirurgie). The anonymous web-based survey was sent to 912 orthopedic surgery residents and 728 medical students on 18 June 2022, who received up to 3 reminders. The survey was then closed on 18 July 2022.

### Statistics

Normally distributed data are expressed as mean ± standard deviation, non-normally distributed data as median (range). To evaluate rankings, an average ranking was calculated for each response option. Different groups were compared using the chi-square test for independence. For small sample sizes, Fisher’s Exact Test was used instead. If the expected data set was 0, small data sets were summarized in order to achieve statistical evaluability. We considered *p*-values of <0.05 to be statistically significant. Data collection and tabulation were performed using Microsoft Excel program (Version 16.78.3). All statistics were calculated with SPSS Statistics^®^ (IBM, Armonk, NY, USA).

## 3. Results

A total of 339 participants completed the survey, representing a response rate of 20.7% from the total population, of whom 130 (38.4%) were medical students.

The cohort consisted of 221 (65.2%) men and 118 (34.8%) women. From the residents surveyed, 16.8% (*n* = 57) were in their first two years of training. Forty-eight (14.2%) respondents reported being in their third or fourth year, and 60 (17.7%) survey respondents were in their fifth or sixth year; 4.7% (*n* = 16) were above the sixth year and 8.3% (*n* = 28) were already junior physicians (Table 1).

Participants answered the question about the department as follows: 172 (51.2%) were in the Department of Orthopedics and Trauma Surgery. Another 50 (14.9%) survey participants were in the Department of Orthopedics and 21 (6.3%) were in the Department of Trauma Surgery; 2.1% (*n* = 7) reported working in private practice, 37 participants (11%) reported another department and 14.6% (*n* = 49) of the respondents worked at none of the above-mentioned departments.

Of all respondents, 75.7% (*n* = 256) indicated Germany as their place of work, 14.6% (*n* = 49) named Austria instead, and 8% (*n* = 27) stated Switzerland. Six residents (1.8%) indicated other countries.

**Figure 1:**
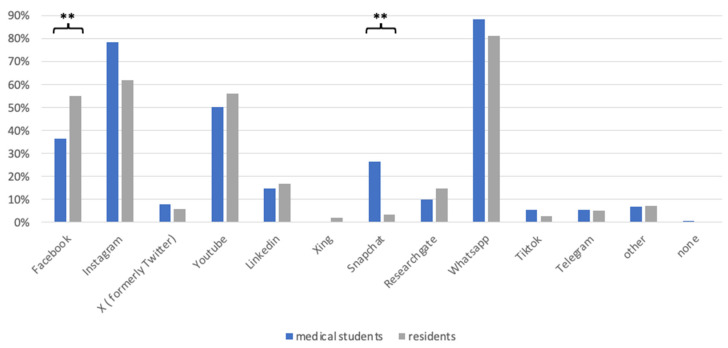
Use of the most common social media platforms in % between medical students and residents (residents + junior specialists). Significance is indicated by ** *p* < 0.05.

The evaluation of social media consumption and the platform used revealed that residents (residents + young specialists; RD) and medical students (MS) use social media once a day or more (87.24%; *n* = 294), with medical students using social media more actively than residents (92.3%; *n* = 120 vs. 78.4%; *n* = 174). The most frequently used platforms in both groups were WhatsApp (MS: 88.4%, *n* = 114; RD: 81%, *n* = 169), followed by Instagram (MS: 78.3%, *n* = 102; RD: 62%, *n* = 130) and YouTube (MS: 50.3%, *n* = 66; RD: 56%, *n* = 116). However, medical students showed a notably higher use of Snapchat (MS: 26.4%, *n* = 34; RD: 3.3%, *n* = 7; *p* = 9.51 × 10^−9^) and TikTok (MS: 5.4% *n* = 6; RD: 2.8% *n* = 7), whereas residents used Facebook more often (MS: 36.4%, *n* = 47; RD: 55%, *n* = 115; *p* = 0.0036, Figure 1). The most frequently used end device was the smartphone at 93.5% (*n* = 316). A total of 20.4% (*n* = 69) of respondents see a high or very high benefit of social media for literature research, whereas 51% (*n* = 174) see little, very little or no benefit at all.

In contrast, 104 participants (32%) see a high or very high benefit for education purposes. In comparison, 113 participants (33%) see little, very little or no benefit. There was no significant difference between the group of medical students and residents in these questions.

Overall, 20% ± 16.23% of the content consumed by medical students and 29% ± 21.93% of the content consumed by residents (*p* = 0.00014) is subject-related content in orthopedics, trauma surgery and sports medicine.

In terms of engagement, 69% (*n* = 234) of participants said they rarely (34%, *n* = 115), occasionally (29.6%, *n* = 100) or frequently (5.6%, *n* = 19) liked, commented on or shared posts. In contrast, 68% (*n* = 229) stated that they never post their own content (text, video, image, link). There were no significant differences between the groups.

The evaluation of whether the participants would follow an influencer (doctor), a medical specialty society or a medical institution on social networks showed that 86% (*n* = 112) of medical students and only 70% (*n* = 146) of residents do so (*p* = 0.000259). Most participants followed content creators with the same specialty (MS: 47% *n* = 56, RD: 51.30% *n* = 102) or the same and a different specialty (MS: 42% *n* = 51, RD: 23.10% *n* = 46; *p* = 0.012).

**Figure 2:**
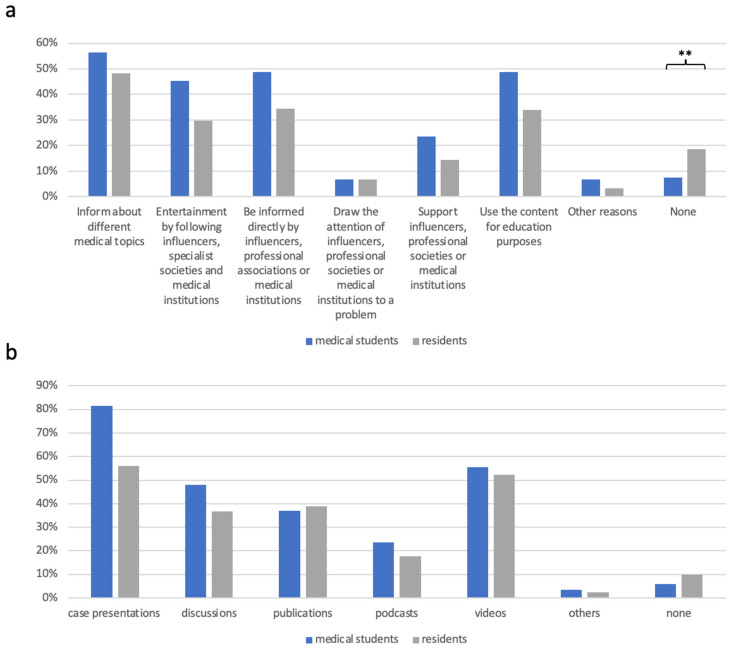
(**a**) Reasons why respondents follow influencers, specialist societies or medical institutions in %. Significance is indicated by ** *p* < 0.05. (**b**) Most beneficial content according to the participants in %.

Reasons why respondents follow influencers, specialist societies or medical institutions are mainly to obtain information about different medical topics (MS: 56.3% *n* = 68, RD: 48.3% *n* = 101), to be informed directly by influencers, specialist societies or medical institutions (MS: 48.7% *n* = 59, RD: 34.4% *n* = 72) and to use the content for education purposes (MS: 48.7% *n* = 59, RD: 34.0% *n* = 71; Figure 2a). Significant differences were found for the answer “None” (MS: 7.6%, *n* = 9; RD: 18.7%, *n* = 39; *p* = 0.0006).

A total of 30% of participants (*n* = 99) rated the subject-related content quality as high to very high, whereas only 16% (*n* = 31) rated it as low or very low.

Regarding which content respondents would benefit most from, the majority reported that they would benefit from case presentations with corresponding images (MS: 81.5% *n* = 98, RD: 56.0% *n* = 117) and videos (MS: 55.5% *n* = 67, RD: 52.2% *n* = 109). While medical students further benefitted from discussions (MS: 48.0% *n* = 57, RD: 36.8% *n* = 77), residents were more interested in publications (MS: 37.0% *n* = 45, RD: 38.8% *n* = 81; Figure 2b).

**Figure 3:**
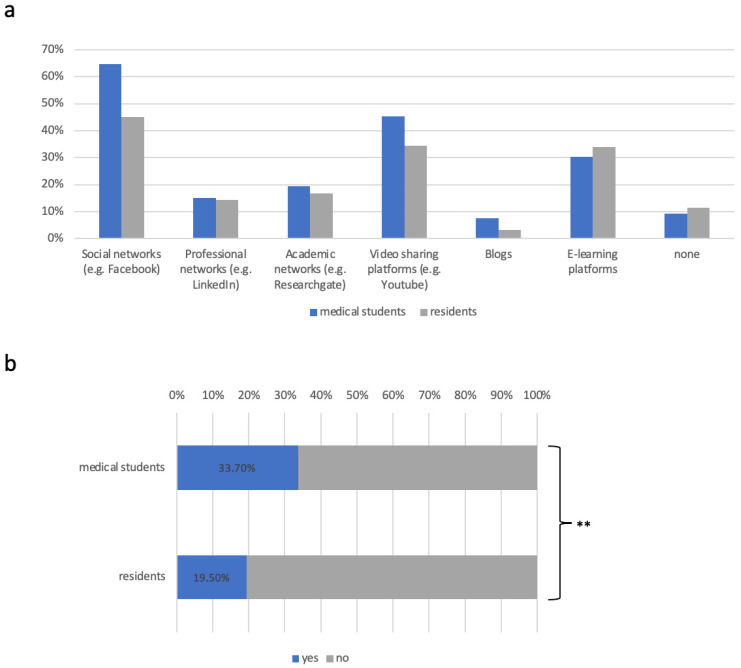
(**a**) Platforms on which respondents wish to see more content. (**b**) Influence of social media on the choice of employer in %. Significance is indicated by ** *p* < 0.05.

The platforms on which respondents would like to see content are “classic” social networks such as Facebook and Instagram (MS: 64.7% *n* = 77, RD: 45.0% *n* = 94; Figure 3a) followed by video-sharing platforms such as YouTube and TikTok (MS: 45.4% *n* = 55, RD: 34.4% *n* = 72) and e-learning platforms (MS: 30.3% *n* = 36 34.0% *n* = 71). One further medium is podcasts. Here, respondents answered that 31% (*n* = 104) already regularly listen to a subject-specific podcast. A further 43% (*n* = 143) could imagine listening to a professional podcast as source of information. Overall, the use of podcasts was more widespread among students (79% *n* = 99) than among residents (76% *n* = 157).

Regarding the question of whether the social media presence had an influence on the choice of employer, a third of medical students (33.6% *n* = 43) answered yes (Figure 3b), whereas it only had an influence for 19.5% of the residents (*n* = 40; *p* = 0.0046).

In total, 15% (*n* = 49) of respondents stated that they had noticed professional contributions that could be considered inappropriate or that violate the patient’s privacy, with one sixth also stating that they were unable to assess this adequately (MS: 21.1% *n* = 26, RD: 14.6% *n* = 30).

## 4. Discussion

Since its advent, social media has indeed taken global communication—and dissemination of information—by storm. The astronomical rise in its user base over the last two decades is evidence of its radical influence over virtually all segments of society, including the realms of education and professional networking among members of the medical community. The purpose of this investigation was therefore to provide a comprehensive assessment of social media usage behavior, preferences and perceptions regarding educational content and professional opportunities of orthopedic and trauma surgery (OTS) residents and medical students in German-speaking countries.

A cross-sectional study showed that orthopedic and trauma practitioners use social media very frequently, such as in our cohort with both residents and medical students [18]. Regarding the different usage platforms, the results of this study support the previous literature.

The findings of our survey provide interesting insights into the OTS social media habitat in the survey’s region. In particular, this study underlines that social media is not just “a thing” or of general interest to the OTS community [16,18]. Indeed, the demographic data represent both similarities and differences to the broader social media user data.

Over these years, a few studies have attempted to quantify social media penetration and to identify the most frequently utilized platforms among orthopedic departments, residencies and fellowships, and to characterize the most accessed content [16,19,20]. Similarly, others have explored the impact of such activity among otolaryngology and radiology residents [21,22,23]. Herein, we add to the limited literature available on social media usage behavior of trainees and medical students in German-speaking countries.

Demographic data showed a diverse cohort, including residents at various levels of training and medial students, with a majority from orthopedic and trauma surgery departments in Germany. Survey results showed the frequency and top platform preferences for social media. The top platform was WhatsApp followed by Instagram and YouTube, suggesting a heavy preference toward instant messaging and visual-based platforms to consume educational content and facilitate professional networking [18,24,25,26]. When stratified by medical students and residents, the medical students showed substantially more social media usage across all major platforms. More specifically, they stated they used more Snapchat, 28% to 10%, respectively, and Tik Tok, 14% to 0%, respectively. This suggests a clear generational trend for platforms that focus on visual and short-form content [3,13,15,27].

It is interesting to note that even while a sizable fraction of participants acknowledged social media’s potential advantages for literary research and educational goals, a noteworthy amount felt that there were few to no advantages. This discrepancy emphasizes the need for more investigation and clarification of the benefits and drawbacks of social media as a resource for orthopedic and trauma surgery professionals’ academic and professional growth.

The results of the study also provided insight into how participants interacted with social media content. Although the majority claimed passive interaction through likes, comments, and shares, a sizable portion stated little to no active content creation, indicating that this cohort uses social media more in line with consumer preferences than creator preferences [14,18,26,28].

This finding emphasizes how crucial it is to use social media platforms to help orthopedic and trauma surgery trainees develop a culture of active involvement and knowledge sharing. Furthermore, there are currently frequent posts that are considered inappropriate or violate the patient’s privacy, which is in line with other studies [11,29]. In addition to privacy concerns, the risk of misinformation remains a critical issue due to the lack of editorial oversight on social media platforms. Integrating social media literacy into residency programs and curricula would help mitigate these risks by training medical students and residents to critically evaluate online information, ensure privacy compliance and improve the overall quality of medical education. In addition, the establishment of small focus groups consisting of administration, residents and healthcare professionals can help to improve privacy, maintain the quality of available information, improve trouble-shooting and identify appropriate solutions for the platforms through their feedback.

The study inquired about the kinds of content that respondents thought were most helpful. Case presentations that included accompanying photos and videos were found to be quite beneficial, highlighting the role that visual aids have in improving learning and comprehension of intricate surgical procedures. Furthermore, the inclination towards content delivery via established social networks such as Facebook and Instagram, together with newer platforms like YouTube and TikTok, highlights the significance of broadening the channels through which content is supplied in order to accommodate the target audience’s changing tastes [17,30,31].

A significant discovery concerns the impact of social media usage on the selection of employers by participants. The increasing significance of institutional and individual online reputations in drawing potential trainees and young professionals is highlighted by the significant percentage of medical students who acknowledged the influence of social media presence in their decision-making process.

Although this study offers insightful information, there are a few important constraints to take into account. First, the low response rate could potentially affect the representativeness of the results, but low response rates are typical for online surveys in the literature [11,14,18].

The survey’s dependence on self-reported data raises the possibility of response bias, potentially overrepresenting frequent users and those with positive views of its educational value. This could skew the results, making social media use seem more prevalent or beneficial than it might be. Therefore, future studies could use random sampling or offer incentives to attract a wider participant base. The primarily German-speaking sample limits the applicability of its conclusions to other groups or regions, due to social media usage being highly variable. Furthermore, it is not possible to draw conclusions about the causes of the correlations between social media usage patterns and educational outcomes that have been found, because the study is cross-sectional.

Finally, this study provides a thorough assessment of how orthopedic and trauma surgery residents as well as medical students in German-speaking countries use social media. The results highlight the various ways that social media contributes to professional networking, education, and career advancement in the field of orthopedic and trauma surgery. Going forward, initiatives to fully utilize social media as a vehicle for professional development and knowledge sharing should be supported by plans to deal with issues with digital literacy, privacy, and the quality of the material provided. Furthermore, future studies should investigate the long-term effects of social media use on professional practices and educational outcomes in this particular field and whether the results shown in this study are consistent in the post-pandemic era.

## 5. Conclusions

Most residents are passive, highly frequent users of social media. They are mainly interested in their focus and follow influencers or institutions. While most users would like to see more content on social media and video-sharing platforms, some note privacy violations or inappropriate content.

## Figures and Tables

**Table 1:** Demographic data of participants.

Characteristics	
Total number of participants	339
Sex	
Male	221 (65.2%)
Female	118 (34.8%)
Year of residency	
medical students	130 (38.4%)
1st–2nd year resident	57 (16.8%)
3rd–4th year resident	48 (14.2%)
5th–6th year resident	60 (17.7%)
>6th year resident	16 (4.7%)
junior specialist	28 (8.3%)
Department	
Department of Orthopedics & Trauma surgery	172 (51.2%)
Department of Orthopedic surgery	50 (14.9%)
Department of Trauma surgery	21 (6.3%)
Private practice	7 (2.1%)
Others	37 (11%)
None	49 (14.6%)
Location	
Germany	256 (75.7%)
Austria	49 (14.6%)
Switzerland	27 (8%)
Others	6 (1.8%)

## Data Availability

The original contributions presented in the study are included in the article/Supplementary Material, further inquiries can be directed to the corresponding author/s.

## Supplementary Materials

The following supporting information can be downloaded at: https://www.mdpi.com/article/10.3390/healthcare12202016/s1, Questionnaire on the use of Social Media among AGA residents and medical students.
