# Supplementary material for: The Use of Social Media in Orthopedic and Trauma Surgery Education: A Cross-Sectional Survey of German-Speaking Residents and Medical Students
Source: Healthcare (Basel). 2024 Oct 10;12(20):2016. doi: 10.3390/healthcare12202016 (PMC11507132; doi:10.3390/healthcare12202016)
Supplement: Supplementary file 1 [file healthcare-12-02016-s001.zip › healthcare-3226230-supplementary.pdf]

Questionnaire on the use of Social Media among AGA residents and medical students

Disclaimer

Sociodemographic Data

1. Which year of training are you in?

   Student
   1-2 year of further training
   3-4 Year of further training
   5-6 year of further training
   >6 year of training
   Specialist rtho/trauma

2. What's your gender?

   Male
   Female

3. At which institution do you work?

   Hospital/department for Orthopaedics and Trauma Surgery
   Hospital/ Department of Orthopaedics
   Hospital/ Department of Trauma Surgery
   Private Practice
   Other
   None

4. Current place of work?

   Germany
   Austria
   Switzerland

Type of social media used

5.

Think about a normal day: How often do you use social networks or social media services?

(Almost) all the time/ constantly
Several times a day
A little once a day
At least once a week
At least once a month
Less often
Never

6.

Which of the following networks do you use at least once a day? (multiple answer)

Facebook
Instagram
Twitter
YouTube
LinkedIn
Xing
Snapchat
Researchgate
Whatsapp
Tiktok
Other
None

7.

More specific: Which social networks do you use most often?
(up to 4 possible answers)

Facebook
Instagram
Twitter
YouTube
LinkedIn
Xing
Snapchat
Researchgate
Whatsapp
Tiktok
Telegram
Other

8.

Which device do you use most often/prefer to use social media on?

Stationary PC
Laptop / notebook / netbook / ultrabook / Macbook
Tablet PC (e.g. Apple iPad, Samsung Galaxy Tab etc.)
Smartphone
Other devices

Social media for educational purposes

9.

How important is social media as a medium for literature research?

Very high
High
Medium
Low
Very little
None at all

10.

How important is social media as a medium for further education purposes?

Very high
High
Medium
Low
Very little
None at all

11.

How much of the content you see on social networks is related to Orthopaedics/ Trauma Surgery/ Sports Medicine?

Very much
A lot
Few
None at all
I don't know
Do not use social networks

Social media consumption

12.

What is it like when others post about Orthopaedics/ Trauma Surgery/ Sports Medicine on social networks? How often do you like, comment on and share other people's specialist posts on social networks?

Frequently
Occasionally
Rarely
Never
Don't know

13.

And how often do you post your own contribution about Orthopaedics / Trauma Surgery / Sports Medicine on social networks, for example a text, a picture, a video or a link related to the subject?

Frequently
Occasionally
Rarely
Never
Don't know

14.

Do you currently follow a doctor (influencer), a professional association or a medical institution on social networks?

Yes
No
I don't know

15.

Are the doctors, specialist societies or medical institutions that you follow mostly

People, institutions and societies that share your focus
People, institutions and societies that have a different focus to you
Both and
I don't know

16.

There are various reasons for following doctors (influencers), specialist societies or medical institutions on social networks. Which of the following reasons apply to you? (multiple answer)

I would like to find out about different medical specialties

I find it entertaining to follow doctors, specialist societies and medical institutions
I like discussing medicine with people
I would like to be informed directly by the doctor, specialist society or medical institution
I would like to draw the attention of the doctor, professional association or medical institution to a problem that concerns me
I would like to express my support for the doctor, professional association or medical institution
I use the content for educational purposes
Other reasons
None of the above
I don't know

17.

How high do you currently rate the quality of content from doctors (influencers), specialist societies or medical institutions in social networks

Very high
High
Medium
Low
Very little
None at all

18.

Which content do you benefit most from? (multiple answer)

Case presentation with corresponding picture
General discussion of topics and overviews
Research publications
Link to podcasts
videos
Other videos
None

F20
Use of podcasts for continuing education purposes

I already use subject-specific podcasts for my continuing education
I could imagine using subject-specific podcasts in the future
I do not see any advantage in using subject-specific podcasts
I generally do not use podcasts

F21
Does social media influence your choice of employer/ Do you use Social media when choosing an employer?

Yes
No

Perceived difficulties of social media use

F22
Have you noticed any professional posts on social media that could be considered inappropriate or violate patient privacy?

Yes
No
I don't know
